# Late Side Effects of Chemotherapy and Radiotherapy in Early Childhood on the Teeth: Two Case Reports

**DOI:** 10.4274/tjh.2017.0216

**Published:** 2018-03-06

**Authors:** Sevcihan Günen Yılmaz, İbrahim Şevki Bayrakdar, Seval Bayrak, Yasin Yaşa

**Affiliations:** 1 Akdeniz University Faculty of Dentistry, Department of Oral and Maxillofacial Radiology, Antalya, Turkey; 2 Eskişehir Osmangazi University Faculty of Dentistry, Department of Oral and Maxillofacial Radiology, Eskişehir, Turkey; 3 Abant İzzet Baysal University Faculty of Dentistry, Department of Oral and Maxillofacial Radiology, Bolu, Turkey; 4 Ordu University Faculty of Dentistry, Department of Oral and Maxillofacial Radiology, Ordu, Turkey

**Keywords:** Pediatric hematologic malignancies, Late side effects, Radiotheraphy and chemotherapy, Teeth, Salivary flow rate

## To the Editor,

Radiotherapy and chemotherapy can generate adverse results during or after the completion of therapy and these treatments can also cause some oral anomalies [1,2,3]. Early and late oral-dental abnormalities have been reported in head and neck cancer patients treated with radiotherapy and chemotherapy. The late side effects of chemotherapy and radiotherapy on the permanent teeth of two patients who had cancer treatment in their early childhood periods are presented here.

**Case 1: **According to the medical history of a 17-year-old female patient who applied to our clinic for routine dental treatment, she had received radiotherapy and chemotherapy due to having Hodgkin lymphoma between the ages of 4 and 5. At the age of 4 years, the patient was admitted to the hospital with an early stage of Hodgkin lymphoma and 4 cycles of the adriamycin, bleomycin, vinblastine, dacarbazine (ABVD) chemotherapy protocol and 20 Gy of neck-region radiotherapy were applied. Complete remission was obtained.

**Figure 1:**
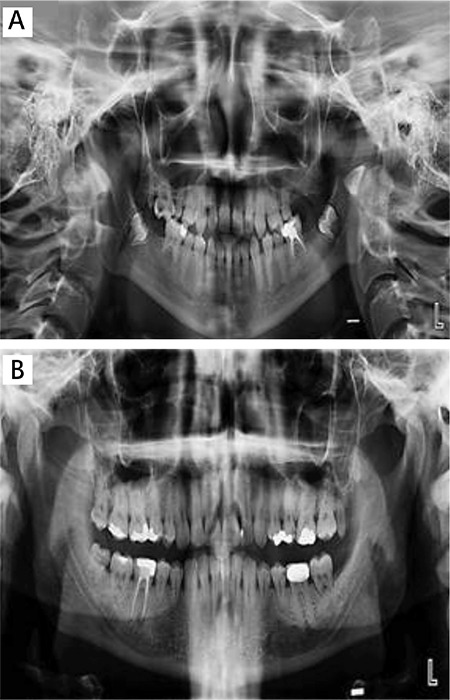
A) Panoramic radiography of case 1; B) panoramic radiography of case 2. A) Panoramic radiography of case 1; B) panoramic radiography of case 2.

In the oral and radiographic examinations, microdontia was found in teeth 11, 15, 17, 21, 25, 36, and 46. The root formations of these teeth were less developed. Due to the lack of germ of teeth 26, 27, 37, and 47, hypodontia was found (Figure 1A). The unstimulated salivary rate was low (0.3 mL/min). The mouth opening was normal.

**Case 2: **According to the medical history of a 24-year-old male patient who applied to our clinic for routine dental treatment, six cycles of ABVD chemotherapy protocol and 30 Gy of radiotherapy to the neck region had been given due to diagnosis of stage III Hodgkin lymphoma between the ages of 7 and 9 years old. Complete remission was achieved.

In oral and radiographic examinations, microdontia was found in teeth 34, 35, 37, 44, 45, 47, and 48 (Figure 1B). The root formations of these teeth were less developed. The unstimulated salivary rate was low (0.3 mL/min). The mouth opening was normal.

Radiotherapy and chemotherapy, which are the main treatments of cancer for young children, can have long-term adverse effects pertaining to the growth and development of orofacial and dental structures.

When pediatric patients are exposed to radiation during the period of tooth development, the formation of dental anomalies such as hypodontia, the cessation of root development, microdontia, taurodontism, temporomandibular joint disorders, malocclusion, and enamel hypoplasia can occur [4,5,6,7,8]. Such problems do not occur in adults.

These treatments may have different effects depending on the dose, the duration of treatment, and the age of the patient [8]. In both of our patients, microdontia due to hypodontia and underdevelopment was observed because dental germs could not be formed [1,2,3,5]. Both patients had low salivary flow rates. This has been observed in previous studies [6]. Both of the patients’ mouth openings were normal. In some studies, limitation of the mouth opening or trismus has been reported.

It has been discovered that chemotherapy and radiotherapy in early childhood have different effects in relation to the doses received in the development stages of the teeth. It is important to inform children who were treated for cancer at early ages and their parents accordingly.
